# COVID-19 and religion: evidence and implications for future public health challenges: Leuconoe Grazia Sisti

**DOI:** 10.1093/eurpub/ckac130.025

**Published:** 2022-10-25

**Authors:** LG Sisti, D Buonsenso, U Moscato, W Malorni

**Affiliations:** 1 Center for Global Health Research and Studies, Università Cattolica del Sacro Cuore, Rome, Italy; 2 National Institute for Health, Migration and Poverty, Rome, Italy; 3 Department of Woman, Child and Public Health, Fondazione Policlinico Universitario A. Gemelli IRCCS, Rome, Italy

## Abstract

**Background:**

Religious and cultural beliefs strongly influence people's attitudes and behaviors that, in turn, may positively or negatively affect both individual and public health. In this regard, we aimed to collect and analyze evidence on the impact of religion in the current COVID-19 pandemic.

**Methods:**

We performed a scoping review investigating both scientific and grey literature available on the topic from the onset of the pandemic to September 2021. Pubmed, Web of Science and Google Scholar were investigated and a hand-search on Google was also performed. Studies dealing with religion and COVID-19 were included and narratively summarized according to topics.

**Results:**

46 articles were included in the review. Predominant topics emerged were 1) religious pilgrimages and rituals worldwide being relevant to COVID-19 outbreaks, especially in the first pandemic wave 2) difficulties to engage the Closed Religious Communities (e.g. Haredi, Amish, etc.) in which community way of life, restrictions in using media and resistance to comply to preventive measures were identified as significant COVID-19 risk 3) COVID-19 unofficial treatments and vaccine hesitancy also supported by concerns on the religious acceptability of vaccine composition or firm interpretation of the Ramadan fasting 4) a fuel of religious discrimination 5) religious communities and leaders strongly trusted in conveying COVID-19 information.

**Conclusions:**

Our findings highlighted how religion has represented both a risk for the spreading of the virus and a precious opportunity to convey evidence-based and culturally-sensitive COVID-19 information engaging people in fighting the pandemic. To be prepared for similar future challenges, scientists, politicians and health professionals need to acknowledge the role that culture and religion have in influencing people's lives to design specific health policies and strategies to ensure that all people are effectively engaged in health production and protection.

**Key messages:**

- Religion has represented both a risk factor for COVID-19 outbreaks and a resource to convey evidence-based information and overcome resistance to implementing COVID-19 preventive measures.
- Health policy should become more sensitive to religious and cultural issues acknowledging the role played by religion in facing complex global health challenges.

